# ‘The end’ – or is it? Emergence of SARS-CoV-2 EG.5 and BA.2.86 subvariants

**DOI:** 10.2217/fvl-2023-0150

**Published:** 2023-09-19

**Authors:** Farid Rahimi, Mohammad Darvishi, Amin Talebi Bezmin Abadi

**Affiliations:** ^1^Research School of Biology, The Australian National University, Ngunnawal & Ngambri Country, Canberra, ACT, 2600, Australia; ^2^Infectious Diseases & Tropical Medicine Research Center (IDTMRC), Department of Aerospace & Subaquatic Medicine, AJA University of Medicinal Sciences, Tehran, 1411718541, Iran; ^3^Department of Bacteriology, Faculty of Medical Sciences, Tarbiat Modares University, Tehran, 14115-111, Iran

**Keywords:** COVID-19, COVID-19 pandemic, COVID-19 vaccine, SARS-CoV-2 variants, variants of concern

Following the initial outbreak of SARS-CoV-2 in 2019 [1], a significant global effort was focused on characterizing the virus and the resulting disease, COVID-19 (searching ‘COVID-19’ on PubMed produced 381,586 entries on 21 August 2023).

Until 19 August 2023, almost 693,681,481 positive cases and an approximate but potentially underreported 6,908,694 deaths due to COVID-19 have been recorded. During the pandemic, the world witnessed the emergence of many viral variants, such as Alpha, Delta, and Omicron, and subvariants, some of which caused further surges of infection [2]. These variants are of considerable concern to public-health experts, clinicians, virologists, epidemiologists and microbiologists because of their potential to be highly transmissible, cause severe disease or render vaccines ineffective against them [3,4]. In May 2023, the WHO announced that COVID-19 was no longer a public-health emergency of international concern thanks to a tangible decrease in hospitalizations, a high level of population immunity and falling numbers of fatalities globally. The legitimacy of the announcement, however, has since been debated due to the emergence of new subvariants and the continued threat posed by COVID-19 [5,6].

Three characteristics of the SARS-CoV-2 variants – immune evasion, transmissibility and disease severity – have driven new infection surges. The mechanisms underlying the emergence of the viral genetic variants at the population and host levels have been discussed [3]; at the outset of the pandemic, many human hosts were naïve and therefore susceptible to SARS-CoV-2, thus, the biological pressure to drive evolution of the variants was minimal. However, later during the pandemic, immunity conferred by vaccination or natural infection and antibody treatments produced unfavorable conditions for the virus, driving the evolution of variants with high transmissibility or the potential to cause more severe disease, as with Delta and Omicron. The most highly transmissible variants outcompeted other variants and propagated among human hosts. RNA genomes of the new SARS-CoV-2 variants have demonstrated a large number of mutations compared with the ancestral strain [7]. The Omicron variant, for example, harbors many mutations that confer a high transmissibility rate and infectivity, immune-evasion capability and the propensity to cause mild disease among vulnerable hosts and spread rapidly among the community [8]. The recent surge of new COVID-19 cases has therefore been spurred on by waning herd immunity (whether induced by natural infection or vaccination) and mutations that have enabled the virus to escape the residual immunity among the population.

More than 66 countries have recorded a newly identified Omicron subvariant called EG.5. In China, this subvariant now accounts for at least 68.3% of COVID-19 cases according to data submitted to GISAID (https://gisaid.org/hcov19-variants/). Considering this, the WHO has upgraded this ‘variant under monitoring’ to a ‘variant of interest’ in early August 2023. EG.5 seems to have initially circulated in China and other Asian regions before spreading to other countries [9]. Only a minority of XBB.1.5 family members carry the mutations that EG.5 harbors; this variant is better equipped to bind the corresponding receptors on human cells and escape antibodies. Unfortunately, two mutations, L455F and F456L, are associated with selection pressures due to monoclonal antibodies administered for treating COVID-19 [9]. Irrespective of the extent of disease severity, the immune-evasion capability and growth advantage of EG.5 are concerning. Though the current number of sequenced samples is low, one new mutation in the viral spike protein has been detected in EG.5. Although the present risk posed by EG.5 to public health is estimated to be low, it cannot be guaranteed to remain a ‘variant of interest’.

Similarly in August 2023, after a surge of COVID-19 cases in the US and the UK, another variant known as BA.2.86 was detected and designated as a ‘variant under monitoring’ because of the many mutations it carries. The main difference between BA.2.86 and EG.5 is that the former has evolved from an earlier Omicron strain named BA.2, which dominated in 2022. The WHO is expected to designate BA.2.86 as a ‘variant of interest’, like EG.5, and possibly even a ‘variant of concern’ in the coming weeks. A high number of hospitalizations due to co-infection by the two variants is likely; however, confirming this may be difficult because previous surveillance systems have been largely downsized or are no longer in operation. Some countries may have unconfirmed cases infected with these variants.

The worrying reports of newly emerged subvariants including EG.5 and BA.2.86 have proven that SARS-CoV-2 variants continue to circulate among susceptible hosts and have shifted our perspective of the pandemic [9]. Having surpassed Alpha, Beta, Gamma and Delta, Omicron and its subvariants have shown that though disease severity is waning, transmissibility is increasing; thus, the virus has evolved a successful strategy to continue to infect many individuals. To understand the processes underlying viral evolution throughout the pandemic, the newly emerged SARS-CoV-2 variants and their interactions with the human immune system must be studied further. Evidence so far suggests that vaccination or natural infection against SARS-CoV-2 mainly prevents severe disease and fatalities. However, to prevent further infection peaks by EG.5 and BA.2.86, the implementation of mask-wearing and other restrictions, at least in indoor settings, may be necessary. One may assume that the COVID-19 pandemic has not ended but merely abated; while the WHO have declared the end to COVID-19 as a public-health emergency, the global community should strive to maintain their capacity to mount an emergency response in the face of any further infection surges [10].
